# Exploring the Mechanisms of Exercise-Induced Hypoalgesia Using Somatosensory and Laser Evoked Potentials

**DOI:** 10.3389/fphys.2016.00581

**Published:** 2016-11-29

**Authors:** Matthew D. Jones, Janet L. Taylor, John Booth, Benjamin K. Barry

**Affiliations:** ^1^School of Medical Sciences, University of New South WalesSydney, NSW, Australia; ^2^Neuroscience Research AustraliaSydney, NSW, Australia

**Keywords:** pain threshold, pain rating, evoked potential, exercise, healthy subjects

## Abstract

Exercise-induced hypoalgesia is well described, but the underlying mechanisms are unclear. The aim of this study was to examine the effect of exercise on somatosensory evoked potentials, laser evoked potentials, pressure pain thresholds and heat pain thresholds. These were recorded before and after 3-min of isometric elbow flexion exercise at 40% of the participant's maximal voluntary force, or an equivalent period of rest. Exercise-induced hypoalgesia was confirmed in two experiments (Experiment 1–SEPs; Experiment 2–LEPs) by increased pressure pain thresholds at biceps brachii (24.3 and 20.6% increase in Experiment 1 and 2, respectively; both *d* > 0.84 and *p* < 0.001) and first dorsal interosseous (18.8 and 21.5% increase in Experiment 1 and 2, respectively; both *d* > 0.57 and *p* < 0.001). In contrast, heat pain thresholds were not significantly different after exercise (forearm: 10.8% increase, *d* = 0.35, *p* = 0.10; hand: 3.6% increase, *d* = 0.06, *p* = 0.74). Contrasting effects of exercise on the amplitude of laser evoked potentials (14.6% decrease, *d* = −0.42, *p* = 0.004) and somatosensory evoked potentials (10.9% increase, *d* = −0.02, *p* = 1) were also observed, while an equivalent period of rest showed similar habituation (laser evoked potential: 7.3% decrease, *d* = −0.25, *p* = 0.14; somatosensory evoked potential: 20.7% decrease, *d* = −0.32, *p* = 0.006). The differential response of pressure pain thresholds and heat pain thresholds to exercise is consistent with relative insensitivity of thermal nociception to the acute hypoalgesic effects of exercise. Conflicting effects of exercise on somatosensory evoked potentials and laser evoked potentials were observed. This may reflect non-nociceptive contributions to the somatosensory evoked potential, but could also indicate that peripheral nociceptors contribute to exercise-induced hypoalgesia.

## Introduction

Exercise relieves pain for many chronic diseases (Hayden et al., 2005; Busch et al., 2007; Fransen et al., 2015), but the mechanisms are poorly understood. The well-described phenomenon of exercise-induced hypoalgesia (EIH) (Naugle et al., 2012) suggests that exercise can reduce pain directly via adjustments at some point(s) in the transduction, transmission and processing of noxious stimuli. Though typically investigated for acute bouts of exercise, there is evidence from cross-sectional (Ellingson et al., 2012; Naugle and Riley, 2014; Lemming et al., 2015; Umeda et al., 2016a) and longitudinal (Anshel and Russell, 1994; Jones et al., 2014) studies that long-term exercise can lead to sustained hypoalgesic effects in healthy adults. EIH is usually measured by obtaining a threshold, tolerance and/or rating of a noxious stimulus and is greatest when mechanical stimuli are used to evoke pain (Koltyn, 2000; Naugle et al., 2012). Moderate effects of exercise on pain are observed when noxious thermal stimuli are used whereas for electrical stimuli, the effect of exercise is smaller still and is less frequently observed (Naugle et al., 2012).

Numerous human and animal investigations have failed to clearly identify the mechanism(s) of EIH. Animal studies have shown that exercise-induced changes in opioids, cannabinoids, catecholamines, and nitric oxide might all contribute to EIH (Galdino et al., 2010a,b, 2015a,b; de Souza et al., 2013; Fuss et al., 2015), whereas human investigations have yielded more equivocal findings (Janal et al., 1984; Kemppainen et al., 1986, 1990; Droste et al., 1991; Koltyn et al., 2014). It has also been proposed that exercise activates an inhibitory arterial baroreceptor mechanism that causes the hypoalgesia (Ring et al., 2008), but there is little evidence to support this (Umeda et al., 2009, 2010). Several studies in healthy individuals have found a positive association between the magnitude of conditioned pain modulation (i.e., pain inhibits pain) and the robustness of EIH (Lemley et al., 2014; Vaegter et al., 2015). However, Ellingson et al. (2014) showed that conditioned pain modulation is likely only a minor contributor to EIH.

Few of these studies in animals, and none in humans, have permitted description of the sites in the nervous system from which EIH arises. For example, animal studies have shown that the effects of drugs acting as or blocking neurotransmitters might arise from several sites in the peripheral and central nervous systems (Galdino et al., 2010a,b, 2014; Sluka et al., 2013; Bobinski et al., 2015; Leung et al., 2016). These include activation of peripheral alpha2 adrenoreceptors and subsequent inhibition of primary afferents (de Souza et al., 2013) or the spinal and supraspinal actions of nitric oxide pathways (Galdino et al., 2015a). Non-invasive neurophysiological techniques have the potential to translate and extend these findings in humans. Laser evoked potentials (LEPs) and somatosensory evoked potentials (SEPs) include components (termed “N2” and “P2”) that scale with the intensity and pain rating of a noxious stimulus (Hu et al., 2014), and occur at latencies consistent with the activation of A-delta or group III afferents (Arendt-Nielsen and Chen, 2003; Truini et al., 2005). The associated laser and electrical stimuli activate, respectively, nociceptors, and nerve axons (Plaghki and Mouraux, 2003; Baumgärtner et al., 2012) and a comparison of LEPs and SEPs provides some insight into the role of the peripheral nociceptor in EIH. That is, if LEPs but not SEPs were to change with exercise, this may indicate a role of the peripheral nociceptor in EIH. However, there are also differences in the ascending and central pathways that contribute to the LEP and SEP, even when a noxious electrical stimulus is used to elicit an SEP (Bromm and Lorenz, 1998; Dowman, 2004).

To the best of our knowledge, only one study has examined LEPs before and after exercise (Friedman et al., 1993) and few studies have examined SEPs before and after exercise (Friedman et al., 1993; Bulut et al., 2003; Micalos et al., 2015). Only one of these studies was designed to investigate EIH (Micalos et al., 2015) and it is unclear to what extent LEPs and SEPs are influenced by exercise; in particular the components of these potentials that are associated with pain. It is well established that the SEP is inferior to the LEP for investigating the activity of nociceptive pathways (Valeriani et al., 2012). In this investigation, the SEP provided data that were used as a complement to the primary measure of the LEP. The SEP in response to noxious stimulation has been used for a similar complementary purpose in a number of recent papers (Arguissain et al., 2015; Ruscheweyh et al., 2015; Rustamov et al., 2016).

The aim of this study was to examine the effect of isometric exercise on LEPs and SEPs to identify changes in excitability of neural pathways accompanying EIH. Pressure pain thresholds (PPTs) and heat pain thresholds (HPTs) were measured to quantify the magnitude of EIH and ratings of pain intensity, pain unpleasantness and anxiety were also recorded throughout the experiments. It was hypothesized that increases in pressure and heat pain thresholds following exercise would be accompanied by a reduction in the amplitude of LEPs and SEPs.

## Methods

### Participants

This study comprised two experiments to examine the influence of acute isometric exercise on SEPs (*Experiment 1*) and LEPs (*Experiment 2*). For each experiment, participants were recruited through advertisements placed on billboards around campus. Eligibility criteria included (1) apparently healthy with no history of chronic pain or chronic disease, (2) between the ages of 18 and 60 years, and (3) absence of a current diagnosis of depression or any other major mood disorder. Sixteen volunteers (age: 22.3 ± 2.9 years (mean ± SD), 9 females) participated in *Experiment 1* and 16 volunteers (age: 24.8 ± 6.0 (mean ± SD), 5 females) participated in *Experiment 2*, of whom 6 had previously participated in *Experiment 1*.

### Procedures

**Figure 1:**
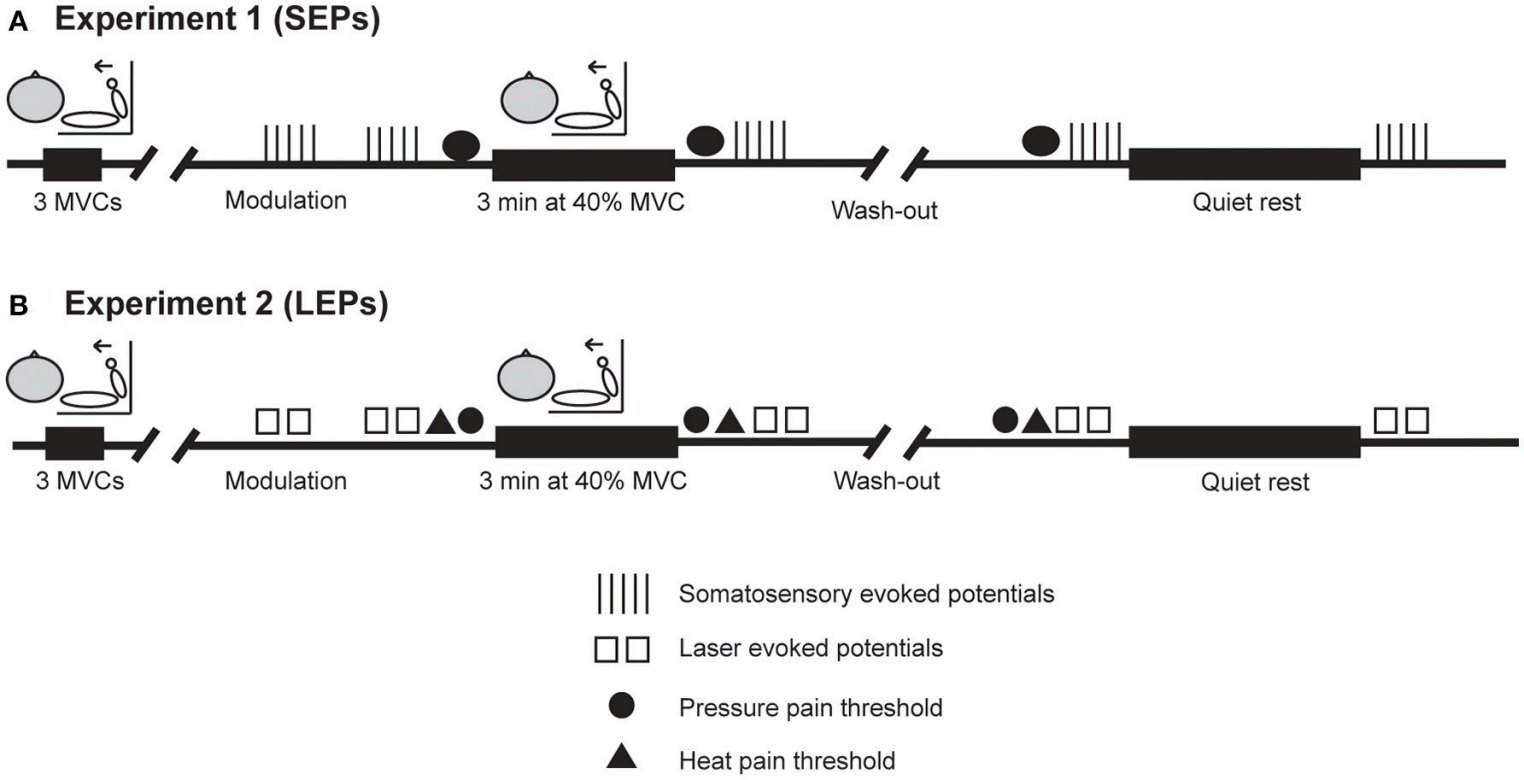
**Experimental procedures. (A,B)** Show the order of procedures in *Experiment 1* (SEPs) and *Experiment 2* (LEPs), respectively, when exercise was performed first. In both experiments, evoked potentials were recorded on five occasions during electrical stimulation (*Experiment 1*) or laser heat stimulation (*Experiment 2*). Pressure pain thresholds were assessed before and after isometric exercise and before quiet rest in each experiment. Heat pain thresholds were assessed before and after isometric exercise and before quiet rest in *Experiment 2* only. A 30 min wash out period was included to ensure possible exercise-induced alterations in pain were gone prior to commencing the next block of evoked potential recordings. This was confirmed by the re-assessment of PPTs (*Experiment 1* and *2*) and HPTs (*Experiment 2* only) prior to the next block of evoked potential recordings. The order of exercise or quiet rest was counterbalanced across participants in each study.

All procedures were approved by the University of New South Wales Human Research Ethics Committee (HC 14065) and conformed to the requirements of the Declaration of Helsinki (2008). Written informed consent was obtained from each participant prior to testing. Before the experiment, participants were asked to abstain from vigorous exercise for 24 h and caffeine for 4 h. Compliance to these requests was confirmed verbally at the start of the session. Each experiment consisted of a single session and lasted approximately 2–3 h. The procedures for each experiment are outlined in Figure 1. Briefly, in each experiment, pain thresholds, pain ratings, and EEG responses to painful stimuli were measured before and after isometric exercise and before and after rest. In *Experiment 1*, evoked potentials during electrical stimulationwere recorded on five occasions (i.e., baseline, before and after rest, and before and after exercise) and pressure pain thresholds were measured before rest as well as before and after exercise. In *Experiment 2*, evoked potentials during laser heat stimulation were recorded on five occasions (i.e., baseline, before and after rest, and before and after exercise) and pressure pain thresholds and heat pain were measured before rest as well as before and after isometric exercise. The order of exercise or rest was counterbalanced across participants in each study. A 30 min wash out period was included to ensure possible exercise-induced alterations in pain were gone prior to commencing the next block of evoked potential recordings. This was confirmed by the re-assessment of PPTs (*Experiment 1* and *2*) and HPTs (*Experiment 2* only) prior to the next block of evoked potential recordings. The 30–50 min required to setup the recording and stimulation equipment ensured that any mild hypoalgesic effect of the preliminary maximal voluntary contractions (MVCs) would have subsided before the collection of evoked potentials (Naugle et al., 2012).

### Electrical stimulation for SEPs (experiment 1)

Electrical stimuli to the digital nerve (1-ms rectangular pulses, frequency 2 Hz, Grass S88, Grass Technologies, Warwick, RI, USA) were delivered through flexible metal ring electrodes attached to the participant's right index finger (cathode proximal). Adhesive electrode gel (Tensive Conductive Adhesive Gel, Parker Laboratories Inc, Fairfield, NJ, USA) and constant current stimulation (Grass CCU1, Grass Technologies, Warwick, RI, USA) was used to maintain consistency of the electrical stimulus throughout the experiment. Each block of stimulation lasted approximately 10 min and consisted of 1000 stimuli (500 at each of two different intensities, delivered randomly). Each block of stimulation comprised five smaller series (100 at each of two different intensities, delivered randomly), separated by 45–60 s.

To determine the intensities used for stimulation, the participant's perceptual threshold was determined by the ascending method of limits and the stimulus intensity corresponding to 1.5 times perceptual threshold was calculated. Then, using a 0–10 numerical and categorical pain rating scale (0 = no pain and 10 = worst possible pain), stimuli were delivered at progressively increasing intensities to determine the intensity that elicited mild pain (3/10) and moderate pain (5–6/10). A short train of stimuli (approximately 10 s) was then delivered at intensities corresponding to 1.5 times perceptual threshold, mild pain and moderate pain, and participants were asked to rate the intensity of pain produced by each short train. This procedure was to ensure that any wind-up effect of repeated stimulation was accommodated in the pain ratings and, if necessary, intensities were adjusted to achieve the desired pain ratings on the scale described above [i.e., moderate pain (5–6/10)].

For the first block of stimulation (termed: “modulation”), stimulus intensities corresponding to mild and moderate pain were used to determine whether SEP amplitude scaled with different intensities of noxious stimulation. For the remaining four blocks of electrical stimulation that occurred before and after rest and before and after exercise, the two stimulus intensities used were non-painful (1.5 times perceptual threshold) and producing moderate pain (5–6/10). During this time, participants were asked to attend to the stimulation occurring at their finger to minimize the possible influence of distraction on the SEP waveform. Following each series of stimulation, participants provided ratings of pain intensity using the scale described above and ratings of pain unpleasantness using a 0–10 pain unpleasantness scale (0 = not at all unpleasant, 10 = most unpleasant imaginable). Anxiety ratings were also obtained after the first, third and fifth series of stimulation within each block on a 0–10 scale (0 = not at all anxious, 10 = worst anxiety imaginable) to account for the possible influence of anxiety on SEP amplitude (Finan et al., 2013).

### Heat stimulation for LEPs (experiment 2)

Throughout the experiment, all persons present in the laboratory wore protective laser goggles for safety. Radiant heat stimuli (30–50 ms duration, 10.6 μm wave length, 3.5 mm beam diameter) were delivered to the dorsal surface of the participant's right hand by a carbon dioxide laser (Synrad 48-1 10 W series, Synrad Inc., Mukilteo, WA, USA). The laser beam was visualized with a He-Ne diode pointer (DP, Synrad Inc,) and laser output was controlled by custom built software (LabVIEW version 9.0, National Instruments, Austin, TX; Spike 2, Cambridge Electronic Design, Cambridge, UK) and a closed-loop stabilization kit (UC-2000, Synrad Inc.). An area of approximately 4 × 4 cm^2^ between the wrist and the base of the 3rd–5th metacarpals was chosen as the target zone for stimulation. To minimize skin damage and reduce the likelihood of nociceptor sensitization or fatigue, successive laser stimuli were delivered to different locations on the hand in a random order such that the same site was never stimulated more than 2–3 times throughout the experiment. The participant's right hand and forearm were placed in an opaque acrylic box lined with laser absorbent cloth and their palm was rested against a padded block with the forearm semi-supinated. Skin temperature was monitored continuously throughout *Experiment 2* using a digital thermode affixed to the base of the participant's right wrist near the anatomical snuffbox.

To familiarize participants with the laser stimulation, a single stimulus was delivered every 5 s at an increasing energy (0.5 mJ/mm^2^) until perceived by the participant (i.e., perceptual threshold). This procedure was repeated two more times and perceptual threshold was recorded as the average of the three trials. Single stimuli were then delivered every 5–6 s at increasing energies to determine the intensity that corresponded to mild pain and moderate pain (i.e., 3/10 and 5/10, respectively). Five blocks of LEPs were recorded throughout the experiment. The first block (termed: “modulation”) comprised 60 total stimuli and was delivered in two separate series of 30 stimuli (15 each of mild and moderate pain, pseudo-random in order, (7–9 s inter-stimulus interval) with approximately 30 s between each series. For the remaining four LEP recording blocks occurring before and after exercise and before and after quiet rest, 30 moderately painful stimuli were delivered in two separate series of 15 (7–9 s inter-stimulus interval and 30 s between series). Ratings of pain intensity, pain unpleasantness and anxiety were recorded after each series. Throughout the recording of LEPs, participants were asked to count the number of stimuli they received at their hand. This was done to ensure attention to the laser stimuli and to minimize the influence of distraction on the LEP waveform.

### EEG recordings

During the recording of evoked potentials in each experiment, participants were seated upright in a dark and quiet room. Silver/silver chloride electrodes (10 mm diameter) were placed along the scalp midline (Cz, Fz and Pz) and left side of the head (C3) and referred to the left earlobe (10–20 International system). A ground electrode was placed across the forehead. Electrode sites were prepared with NuPrep (Weaver and Company, Aurora, CO, USA), abraded with sandpaper (CIVCO, Kalona, IA, USA) and adhered with Ten20 conductive paste (Weaver and Company) and tape. A small plastic probe was used to further agitate the skin as necessary to reduce impedance at each electrode to less than 5000 ohms. Contact impedance was monitored and recorded throughout each experiment using built-in features of the EEG amplifiers and kept below 5000 ohms.

The EEG signal was amplified 5000x for SEPs and 1000x for LEPs (NL844 and NL820A, Digitimer NeuroLog System, Hertfordshire, England), filtered (0.1 Hz–2 kHz, NL144, and NL135/6,) and collected on a computer (Spike 2 and Micro1401 mkII, Cambridge Electronic Design) at 5000 samples per second. Electrooculography (EOG) was recorded using 6 mm gold cup electrodes placed above and below the left eye and was monitored continuously by one of the experimenters. When necessary, participants were guided to relax to maintain the baseline stability of these signals.

### Pressure pain thresholds

In each experiment, PPT was assessed over the biceps brachii and first dorsal interosseous muscles. All measurements were made on the right side of the body. Two practice trials were performed on the left biceps brachii prior to testing to familiarize the participant with the procedure. The rubber-tipped probe of a handheld algometer (Wagner Force 10 FDX-25, Wagner Instruments, Greenwich, CT) was applied perpendicularly to the participant's skin and the force was increased gradually at a rate of approximately 1 kg/s. Participants were instructed to give a verbal command of “stop” when the sensation of pressure turned to pain. This procedure was repeated two more times for a total of three measurements per site. Pressure pain threshold was recorded as the average of these three measurements.

### Heat pain threshold

In *Experiment 2* only, brief laser stimuli (20 ms) were delivered at a frequency of 5.55 Hz at a progressively increasing intensity (approximately 0.5 mJ/mm^2^/s) to the dorsal surface of the participant's right hand. Participants were instructed to give a verbal command of “stop” as soon as the stimulation became painful. This procedure was repeated three more times for a total of four measurements and HPT was recorded as the average of these four measurements. The frequency, duration and intensity of this stimulation was chosen so that HPTs were obtained over a similar time period and evoked a similar sensation to PPTs (i.e., a continuous sensation, gradually increased in intensity until painful). After several experimental sessions, an assessment of HPT at the right forearm was added (*n* = 13), akin to measuring PPTs at the hand and upper arm. The forearm was used because of safety concerns about directing the laser at the upper arm, nearer the face and eyes.

### Isometric exercise

In each experiment, participants were seated upright in an adjustable chair with their forearm neutral and rested on a padded support parallel to the floor. Participants grasped, with their right hand, a custom built device that was instrumented with a force transducer (SBO-100, Transducer techniques, Temecula, CA, USA). The force transducer measured the medially-directed force of elbow flexion via a hand grip. The signal was amplified and filtered (200x, 0–100 Hz, NL109, Digitimer Neurolog System) and was recorded at 200 Hz (Spike 2 software). At the start of the experiment, participants performed three MVCs, each separated by 60 s, and the highest value of these three attempts was recorded as MVC. For the experimental isometric exercise task, a 3-min sustained contraction at 40% of MVC was performed. During this time, participants were asked to match the target force displayed on a monitor and to provide ratings of perceived exertion (RPE) every 30 s on a Borg 0–10 scale.

### Quiet rest and wash out

In each experiment, a period of quiet rest (approximately 4 min) was included to correspond to the time it took to set up and perform the isometric exercise task. During quiet rest, participants remained seated and relaxed but were allowed to talk to the experimenters. A wash out period of approximately 30 min was also included to ensure any hypoalgesic effect of the exercise would have subsided before the collection of the next series of evoked potentials.

## Data processing and statistical analysis

### EEG and evoked potential analysis

Evoked potentials were extracted using computer software (Spike 2) by averaging the EEG signal following the electrical or laser stimuli. Prior to averaging, signals were visually inspected and stimulation triggers were removed when artifacts were apparent in the EOG recording. On average (mean ± SD), 10 ± 15 triggers were removed prior to averaging for SEPs and 2 ± 3 triggers were removed prior to averaging for LEPs. A digital filter (Finite Impulse Response, 30-Hz lowpass) was applied to the EEG recordings before processing the LEPs. No digital filtering was necessary for the SEP recordings. In *Experiment 1*, EEG data were divided into 550-ms epochs, each lasting from −50 to +500 ms with respect to stimulus onset. The N2P2 component of the SEP, which is thought to reflect a cortical response to activation of nociceptive afferents, was analyzed at each of the 4 EEG sites according to the procedures described by Luck (2005). The baseline signal was calculated as the root mean square amplitude in the 50 ms prior to each electrical stimulus. The N2P2 onset was quantified as the time in which the EEG signal was 1 standard deviation above baseline (negative polarity) 100–300 ms after stimulus onset. N2P2 amplitude was calculated as the difference between the N2 and P2 peak amplitudes which were measured from 100–300 ms (N2) to 150–400 ms (P2). N1 amplitude, which reflects cortical arrival of a volley through fast conducting afferents, was calculated for the SEPs as the difference between the baseline signal and the peak negative polarity 20–60 ms after stimulus onset. In *Experiment 2*, EEG data were divided into 1050-ms epochs, each lasting from −50 ms to +1000 ms with respect to stimulus onset. The N2P2 component was analyzed at each of the 4 EEG sites as described above but for slightly later time periods (N2: 150–500 ms; P2: 200–550 ms) to account for the longer stimulus duration and activation of nociceptors by the laser heat stimulus.

### Sample size calculations

Each participant was tested before and after exercise intervention and rest, meaning that they acted as their own control, which allowed changes to be reliably detected with relatively few participants. Notably absent from most evoked potential studies (Larson and Carbine, 2016), sample size calculations were performed using G^*^Power (version 3.1.9.2) (Faul et al., 2007). These were made for the pain threshold and evoked potential measures on the basis of changes observed in previous investigations. For the effect of acute exercise on pressure pain thresholds, we estimated a mean change of 0.30 ± 0.16 kg/cm^2^ (Naugle et al., 2014a), corresponding to a large effect size (*d* = 1.87). On this basis, it was estimated that a sample size of ≥5 participants was required to detect exercise-induced hypoalgesia using pressure pain thresholds with a repeated measures test, 80% power and alpha of 0.05. Very limited data were available to estimate the effect of exercise on pain-related evoked potentials. Small to moderate effect sizes were anticipated on the basis of reported changes in the P1 and P2 amplitude of somatosensory evoked potentials after acute exercise (Bulut et al., 2003) and from changes in the nociceptive flexion reflex following exercise (Guieu et al., 1992). A sample size of 8–12 was computed to be required to detect a small-moderate effect size of change in the evoked potentials. A target sample of 14–16 participants was planned for each study to provide more power and precision, and to account for the possibility of participant drop out.

### Statistical analysis

Descriptive statistics were calculated using the IBM Statistical Package for Social Sciences (version 22, Chicago, IL, USA). Differences in pressure and heat pain thresholds were examined using a repeated measures ANOVA and differences in pain ratings and evoked potential amplitude were tested with a two way repeated measures ANOVA (time: pre, post; condition: rest, exercise). To compare changes in PPTs and evoked potential amplitude between *Experiment 1* and *2*, a repeated measures ANOVA with time (pre, post) and condition (rest, exercise) as within subject factors and the experimental condition (SEP–*Experiment 1* or LEP–*Experiment 2*) as a between subjects factor was used. Normality of the data was assessed using the Shapiro-Wilk Test. Greenhouse-Geisser corrections were used if sphericity was violated. To identify sources of differences revealed by the ANOVA, paired sample *post-hoc t*-tests were conducted with alpha set at 0.05 and the *p*-values for the *t*-tests multiplied by the number of comparisons within the ANOVA model. Effect sizes (unbiased Cohen's *d*) and 95% confidence intervals (CIs) were also calculated to aid comparisons between different measures and between *Experiment 1* and *2*. Effect sizes (ES) were interpreted as small (0.2), medium (0.5) or large (0.8) (Cohen, 1988). The unbiased Cohen's *d* was used because it is a conservative estimate that avoids overestimation of effect sizes when using small sample sizes (i.e., *n* < 30). The 95% CIs of the effect size were calculated using a non-central *t* distribution (Cumming, 2012). Except where stated, values are reported as the mean and 95% CI.

## Results

### Isometric exercise

All participants were able to maintain the target force during the 3-min contraction. The average RPE (mean ± SD) at the end of the contraction was 7.9 ± 1.4 and 9.2 ± 1.2 in *Experiment 1* and *2*, respectively. This corresponded to a perceived effort between “very hard” and “very, very hard (maximal).” The average RPE at the end of isometric exercise was higher in *Experiment 2* than *Experiment 1* [*d* = 0.93 (0.21–1.68), *p* = 0.01].

### Pressure pain thresholds

**Figure 2:**
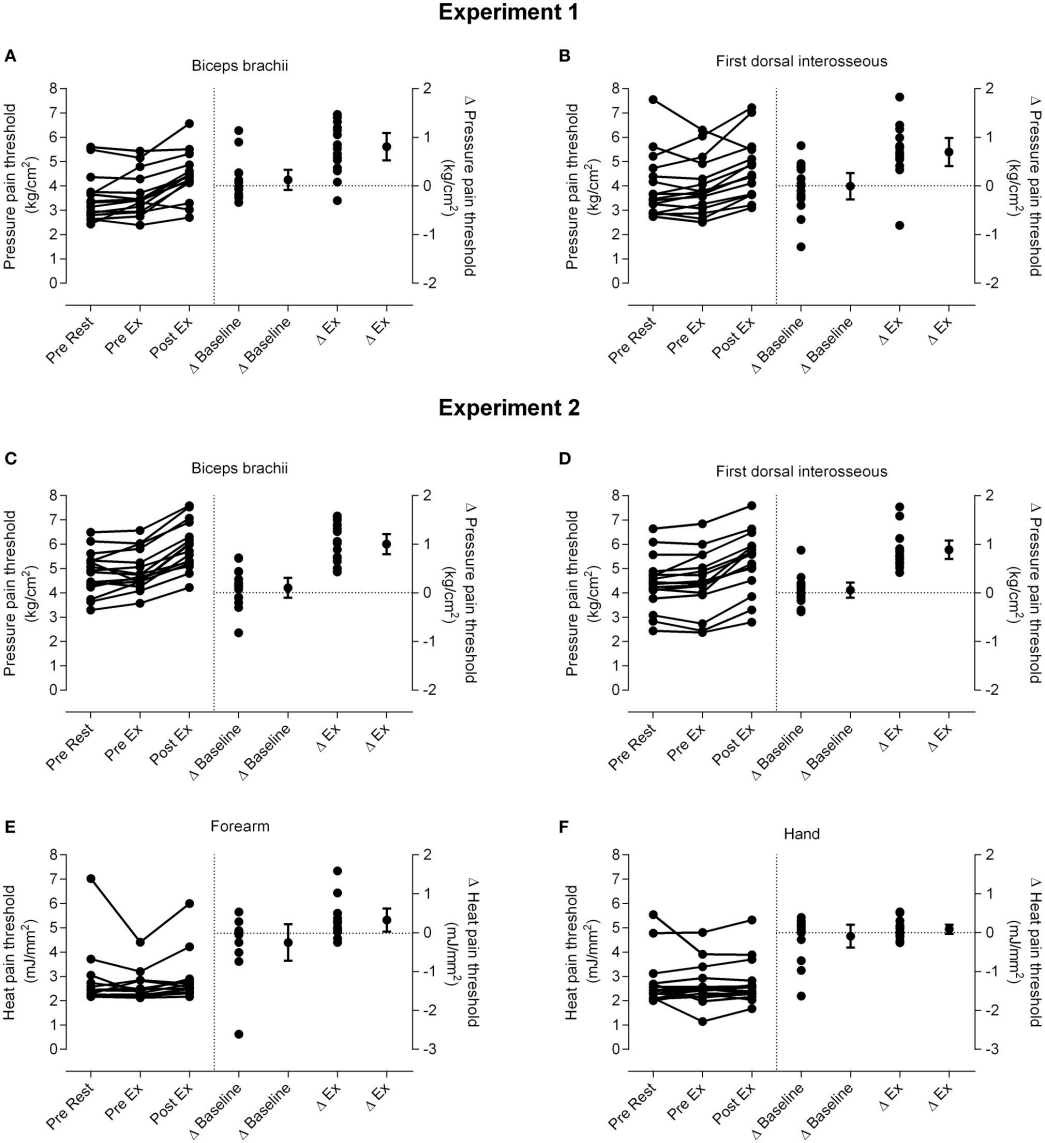
**Changes in pain threshold**. Individual data for pressure pain thresholds (PPTs; left side of vertical dotted line) and the differences in PPTs for individual participants and the group (mean and 95% confidence interval; right side of vertical line) at m. biceps brachii in *Experiment 1* **(A)** and *Experiment 2* **(C)** and m. first dorsal interosseous in *Experiment 1* **(B)** and *Experiment 2* **(D)**. Individual data for heat pain thresholds (HPTs; left side of vertical dotted line) and the differences in HPTs for individual participants and the group (mean and 95% confidence interval; right side of vertical line) at the forearm **(E)** and hand **(F)** in *Experiment 2* are also shown. Δ baseline is the difference between the pre rest and pre exercise measures and Δ ex (exercise) is the difference between the pre exercise and post exercise measures. Data to the left of the vertical dotted line are plotted against the left-hand y-axis and data to the right of the vertical dotted line are plotted against the right-hand y-axis.

Data for PPTs are presented in Figure 2. ANOVA indicated a significant effect of time for PPT over the biceps brachii [*Experiment 1: F*_(2, 30)_ = 33.06, *p* < 0.001; *Experiment 2: F*_(2, 30)_ = 69.42, *p* < 0.001] and first dorsal interosseous muscles [*Experiment 1: F*_(1.15, 17.19)_ = 10.53, *p* = 0.008; *Experiment 2: F*_(2, 30)_ = 68.28, *p* < 0.001]. *T*-tests showed there was a large and significant effect of isometric exercise on increasing PPT over biceps brachii [*Experiment 1*: 24.3 ± 17.6% increase (mean ± SD), *d* = 0.84 (0.45–1.3), *p* < 0.001; *Experiment 2:* 20.6 ± 8.3% increase (mean ± SD), *d* = 0.99 (0.59–1.49), *p* < 0.001] and a moderate and significant effect on increasing PPT over first dorsal interosseous [*Experiment 1*: 18.8 ± 11.6% increase (mean ± SD), *d* = 0.57 (0.28–0.9), *p* < 0.001; *Experiment 2:* 21.5 ± 11.1% increase (mean ± SD), *d* = 0.67 (0.42–0.98), *p* < 0.001], whereas PPTs at both muscles were similar before rest and before exercise (range of mean change = 0.9–4.7% increase, all *d* < 0.13 and *p* > 0.32; Figure 2). The magnitude of EIH, as quantified by the increase of PPTs, was similar between the experiments at biceps brachii [*Experiment 1*: 0.81 ± 0.53 kg/cm^2^ (mean ± SD); *Experiment 2*: 1.00 ± 0.39, *p* = 0.25] and first dorsal interosseous [*Experiment 1*: 0.70 ± 0.55 (mean ± SD); *Experiment 2*: 0.89 ± 0.35; *p* = 0.26].

### Heat pain thresholds

Data for HPTs (*Experiment 2*) are presented in Figure 2. There was no significant effect of time on HPTs over the forearm [*F*_(2, 24)_ = 2.23, *p* = 0.10] or hand [*F*_(1.27, 19.05)_ = 0.39, *p* = 0.74].

### Electrical and laser heat stimulation for the evoked potentials

The average electrical stimulus intensities used to elicit mild and moderate pain during modulation in *Experiment 1* were 23.5 ± 12.0 mA and 43.0 ± 21.5 mA (mean ± SD), respectively. For the remaining four blocks of stimulation, the intensities that corresponded to 1.5 times perceptual threshold and moderate pain were 3.0 ± 0.6 and 42.0 ± 20.0 mA (mean ± SD), respectively. In *Experiment 2*, the average stimulus intensities used to elicit mild and moderate pain were 13.45 ± 8.75 and 19.44 ± 6.13 mJ/mm^2^ (mean ± SD), respectively. Skin temperature remained stable throughout the experiment for each participant [range 1.51 ± 0.51°C, (mean ± SD)]. Laser stimulation caused small red spots to appear on the skin of all participants during the session. Within 1–2 days these darkened and then disappeared after 2–3 weeks. This was never reported as painful, but was sometimes reported as being itchy. All participants were informed of this common effect of carbon dioxide lasers prior to giving their informed consent.

### Evoked potential waveforms

**Figure 3:**
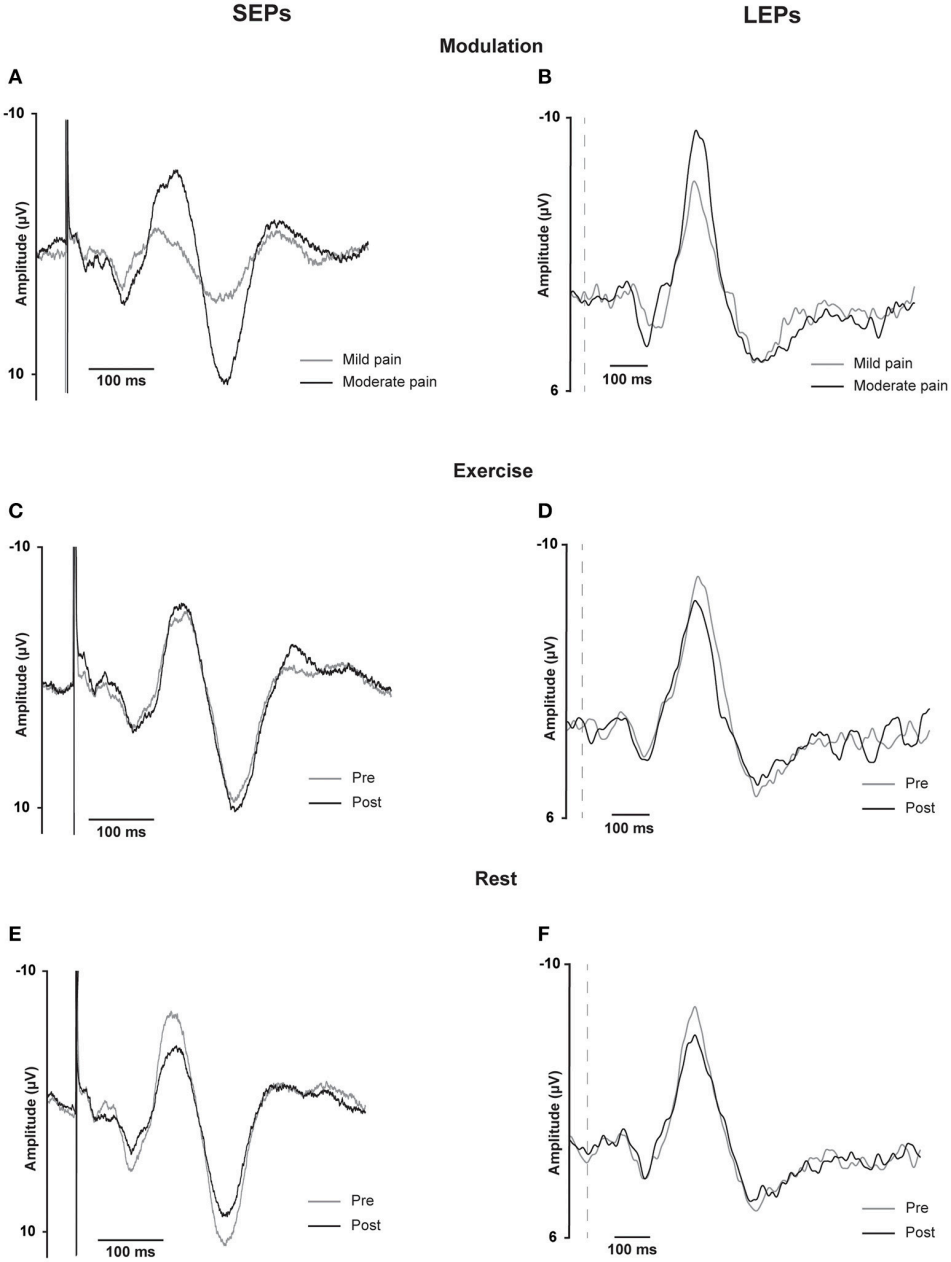
**SEP and LEP grand averages for Cz**. Somatosensensory evoked potentials recorded at Cz from 16 participants in *Experiment 1* (SEPs, **A,C,E** on the left) and laser evoked potentials recorded at Cz from 16 participants in *Experiment 2* (**B,D,F** on the right). These traces are the grand averages across participants of individual waveform averages from approximately 500 stimuli for the SEPs and from approximately 30 stimuli for the LEPs. Data are shown for SEPs and LEPs recorded during the modulation test in response to different intensities of stimulation **(A,B)** or immediately before and after exercise **(C,D)** or rest **(E,F)**. For the modulation test, two stimulus intensities corresponding to either mild or moderate pain were randomly presented within the same sequence of 5 test blocks. For the SEPs, data are shown for 50 ms before and 450 ms following the stimulus onset; the stimulus artifact is visible on each plot and has been truncated for the illustration. For the LEPs, data are shown for 50 ms before and 950 ms following the stimulus onset; the vertical dashed lines represent stimulus onset.

**Figure 4:**
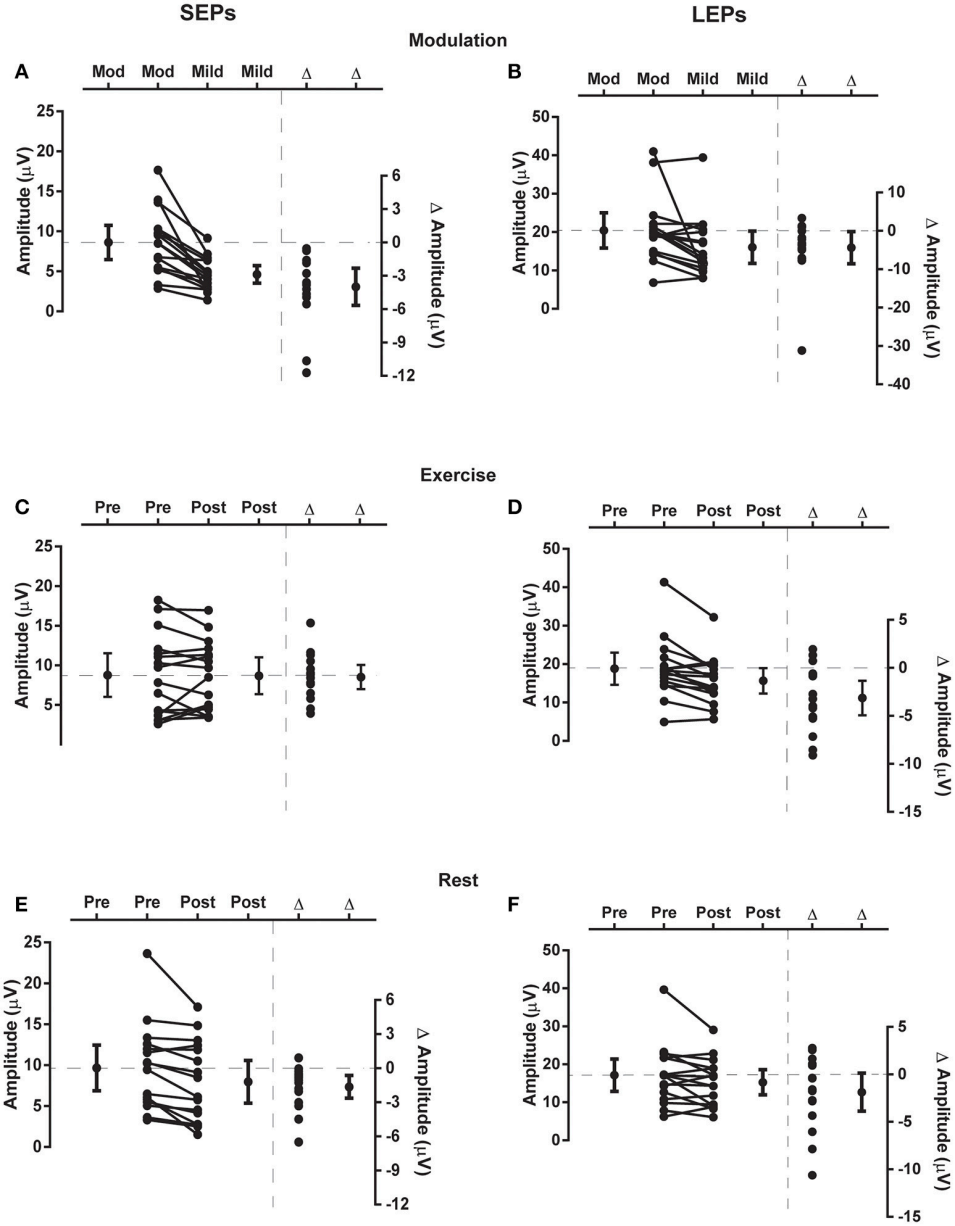
**Changes in evoked potential N2P2 amplitude**. Each panel presents individual and group data (mean and 95% confidence interval) for the N2P2 evoked potential amplitude to the left side of vertical dashed line and individual and group differences (Δ; mean and 95% confidence interval) in evoked potential amplitude to the right side of vertical dashed line. SEP data from *Experiment 1* are in the left panels (SEPs, **A,C,E**) and LEP data from *Experiment 2* are in the right panels (LEPs, **B,D,F**). **(A,B)** Responses to mild and moderate (mod) pain stimuli recorded in the modulation blocks. **(C,D)** Responses recorded before (pre) and after (post) exercise. **(E,F)** Responses recorded before and after a period of rest. In each of these plots the zero-difference level on the right-hand y-axis is aligned to the group mean for the reference condition of moderate stimulation intensity **(A,B)**, pre-exercise **(C,D)** or pre-rest **(E,F)**. Data to the left of the vertical dashed line are plotted against the left-hand y-axis and data to the right of the vertical dashed line are plotted against the right-hand y-axis.

Grand average evoked potential waveforms from Cz are shown in Figure 3 and individual and group data are shown in Figure 4. Summary data from Cz for the N2P2 evoked potential amplitude and onset latency for each condition in *Experiment 1* and *2* are shown in Tables 1, 2, respectively.

**Table 1:** **Mean ± SD peak-to-peak amplitude (μV) and onset latency (ms), and effect size of the change, for the SEP N2P2 waveform at Cz in Experiment 1**.

	**Modulation (mild)**	**Modulation (moderate)**	**Δ Modulation**	**Pre-rest**	**Post-rest**	**Δ Rest**	**Pre-exercise**	**Post-exercise**	**Δ Exercise**
**N2P2**									
Amplitude	4.63±2.07	8.61±4.03	1.18 (0.58 to 1.89)^†^	9.63±5.26	7.96±4.90	−0.32 (−0.55 to −0.11)^*^	8.77±5.16	8.68±4.38	−0.02 (−0.23 to 0.19)
Onset	108.2±16.0	114.9±12.2	0.45 (0.11 to 0.83)^*^	125.1±19.0	121.6±20.3	−0.17 (−0.59 to 0.24)	118.7±18.6	117.4±15.2	−0.07 (−0.66 to 0.51)
**N1**									
Amplitude	1.45±0.98	1.39±0.93	−0.07 (−0.41 to 0.27)	1.82±1.31	0.87±0.4	−0.93 (−1.72 to −0.21)^*^	1.42±1.16	1.76±1.19	0.27 (−0.05 to 0.61)
Onset	41.5±14.7	35.8±13.1	−0.39 (−1.03 to 0.22)	46.5±12.0	33.1±11.2	−1.09 (−1.91 to −0.35)^*^	42.1±14.5	45.9±13.2	0.28 (−0.29 to 0.87)

*The baseline-to-peak amplitude (μV) and onset latency (ms) of the SEP N1 waveform, and effect size of the change, is also presented*.

*The gray columns show the effect size and 95% confidence interval for the change in each of these waveform components for each condition (modulation, rest and exercise). Unless specified, all data relate to responses to moderately painful stimulation*.

* p < 0.02;

† *p < 0.001*.

**Table 2:** **Mean ± SD peak-to-peak amplitude (μV) and onset latency (ms), and effect size of the change, for the LEP N2P2 waveform at Cz in Experiment 2**.

	**Modulation (mild)**	**Modulation (moderate)**	**Δ Modulation**	**Pre-rest**	**Post-rest**	**Δ Rest**	**Pre-exercise**	**Post-exercise**	**Δ Exercise**
Amplitude	16.05±7.90	20.45±8.61	0.51 (0.02 to 1.03)^*^	17.13±7.96	15.26±6.12	−0.25 (−0.53 to 0.02)	18.80±7.88	15.66±6.23	−0.42 (−0.72 to −0.16)^†^
Onset	252.9±39.9	233.8±41.3	−0.44 (−0.93 to −0.02)	223.3±44.3	217.2±48.8	−0.10 (−0.38 to 0.17)	237.6±41.3	235.4±42.2	−0.05 (−0.29 to 0.18)

*The gray columns show the effect size and 95% confidence interval for the change in each of these waveform components for each condition (modulation, rest, and exercise)*.

* p < 0.05;

† *p < 0.01*.

### Modulation of SEPs and LEPs in response to different intensities of stimulation

Moderate-large and significant effects of higher stimulus intensity on increasing SEP amplitude (*p* < 0.001) and onset latency (*p* = 0.011) were observed for the N2P2 (Figures 3A, 4A, Table 1). In contrast, the amplitude (*p* = 0.69) and onset latency (*p* = 0.21) of the N1 potential of the SEP was unchanged for the mild and moderate intensities of stimulation (Table 1). For the LEPs, there was a moderate and significant effect (*p* = 0.041) of higher stimulus intensity on increasing N2P2 amplitude (Figures 3B, 4B, Table 2) but no effect on the LEP N2P2 onset latency (*p* = 0.06; Table 2).

### Effect of exercise and rest on SEPs

For the exercise and rest conditions in *Experiment 1*, two stimulus intensities corresponding to either moderate pain or non-painful stimulation at 1.5 × perceptual threshold were randomly presented within the same sequence of 5 test blocks. The reported N1 and N2P2 responses were elicited by the stimulus intensity that caused moderate pain, while stimulation at 1.5 × perceptual threshold did not consistently yield measurable responses. For SEP N1 amplitude during moderately painful stimulation, there was no significant effect of time [*F*_(1, 15)_ = 2.14, *p* = 0.16] or condition [*F*_(1, 15)_ = 2.47, *p* = 0.14], but a significant time × condition interaction [*F*_(1, 15)_ = 6.99, *p* = 0.018] was observed. SEP N1 amplitude was significantly lower after quiet rest (*p* = 0.024) but not exercise (*p* = 0.18; Table 1). Similarly, for SEP N1 onset, there was no significant effect of time [*F*_(1, 15)_ = 4.12, *p* = 0.06] or condition [*F*_(1, 15)_ = 1.43, *p* = 0.25], but a significant time x condition interaction [*F*_(1, 15)_ = 7.75, *p* = 0.014] was observed. SEP N1 was earlier after quiet rest (*p* = 0.008) but not exercise (*p* = 0.66; Table 1).

For SEP N2P2 amplitude during moderately painful stimulation, there was no significant effect of condition (*p* = 0.85), but a significant effect of time [*F*_(1, 15)_ = 7.39, *p* = 0.016] and a significant time x condition interaction [*F*_(1, 15)_ = 4.71, *p* = 0.047] were observed. SEP amplitudes were significantly lower after quiet rest (*p* = 0.006) but not exercise (*p* = 1; Table 1, Figures 4E,C, respectively). Comparison between the changes in SEP N2P2 amplitude in the exercise and quiet rest conditions showed a moderate to large effect, which was significant [*d* = −0.77 (−1.59 to −0.01), *p* = 0.047]. There was no significant effect of time, condition, nor a time × condition interaction on N2P2 onset (all *p* > 0.19). The pattern of change in the SEP N2P2 waveform at the other EEG sites (Fz, Pz, and C3) was similar to that of Cz but generally smaller in magnitude (Table 3).

**Table 3:** **Mean ± SD peak-to-peak amplitude (μV) and onset latency (ms), and effect size of the change, for the SEP N2P2 waveform at Fz, Pz, and C3 in Experiment 1**.

	**Modulation (mild)**	**Modulation (moderate)**	**Δ Modulation**	**Pre-rest**	**Post-rest**	**Δ Rest**	**Pre-exercise**	**Post-exercise**	**Δ Exercise**
**Fz**									
Amplitude	2.92±1.02	4.53±1.40	1.25 (0.71 to 1.90)^*^	5.09±2.05	4.26±1.98	−0.39 (−0.88 to 0.06)	4.38±2.02	4.52±1.59	0.08 (−0.31 to 0.48)
Onset	120.0±32.4	112.2±22.1	−0.27 (−1.08 to 0.52)	113.3±19.1	110.1±22.8	−0.14 (−0.60 to 0.31)	113.7±24.9	111.3±22.2	−0.09 (−0.46 to 0.27)
**Pz**									
Amplitude	3.66±1.53	5.66±2.61	0.91 (0.42 to 1.47)^*^	6.20±2.86	5.71±3.39	−0.15 (−0.46 to 0.15)	6.19±3.47	5.89±3.07	−0.08 (−0.27 to 0.10)
Onset	110.4±16.1	122.4±27.5	0.51 (−0.35 to 1.40)	108.6±25.6	116.1±21.3	0.30 (−0.23 to 0.85)	118.0±26.3	123.9±29.6	0.20 (−0.26 to 0.67)
**C3**									
Amplitude	3.41±1.58	5.10±2.11	0.86 (0.39 to 1.41)^*^	5.79±2.55	4.79±2.50	−0.38 (−0.61 to −0.17)^*^	5.15±2.55	5.37±2.46	0.09 (−0.10 to 0.28)
Onset	102.8±8.8	103.5±8.0	0.08 (−0.33 to 0.50)	103.1±14.3	106.9±14.5	0.25 (−0.40 to 0.92)	108.7±17.2	105.2±19.1	−0.18 (−0.84 to 0.46)

*The gray columns show the effect size and 95% confidence interval for the change in each of these waveform components for each condition (modulation, rest and exercise). Unless specified, all data relate to responses to moderately painful stimulation*.

* *p < 0.001*.

### Effect of exercise and rest on LEPs

There was a significant effect of time [*F*_(1, 15)_ = 13.66, *p* = 0.002] but not condition [*F*_(1, 15)_ = 1.47, *p* = 0.24] nor a time × condition interaction [*F*_(1, 15)_ = 1.18, *p* = 0.29] on LEP amplitude. In accord with the time effect, mean LEP amplitudes were lower after rest and exercise, but the reduction was only significant after exercise (*p* = 0.004; Table 2 and Figures 4D,F). While the mean change score in the LEP following exercise was larger in magnitude than that following rest, the difference between the changes in the two conditions was small and non-significant [*d* = 0.33 (−0.30 to 0.99), *p* = 0.29]. For LEP onset, there was no significant effect of time, condition, or a time × condition interaction (all *p* > 0.13). The pattern of change in the LEP N2P2 waveform at the other EEG sites (Fz, Pz, and C3) was similar to that of Cz but was generally smaller in magnitude (Table 4).

**Table 4:** **Mean ± SD peak-to-peak amplitude (μV) and onset latency (ms) of the LEP N2P2 waveform at Fz, Pz, and C3 in Experiment 2**.

	**Modulation (mild)**	**Modulation (moderate)**	**Δ Modulation**	**Pre-rest**	**Post-rest**	**Δ Rest**	**Pre-exercise**	**Post-exercise**	**Δ Exercise**
**Fz**									
Amplitude	9.43±3.98	13.29±5.93	0.72 (0.17 to 1.34)^*^	11.28±4.08	10.91±4.60	−0.08 (−0.40 to 0.23)	13.35±4.91	11.02±4.30	−0.48 (−1.06 to 0.07)
Onset	259.6±52.3	237.0±42.9	−0.45 (−1.12 to 0.19)	221.5±60.9	239.8±60.2	0.29 (−0.31 to 0.90)	231.4±61.1	243.5±46.8	0.21 (−0.41 to 0.85)
**Pz**									
Amplitude	13.79±6.56	17.45±6.91	0.51 (0.14 to 0.93)^†^	14.95±6.86	13.12±5.30	−0.28 (−0.63 to 0.04)	16.87±7.60	13.27±6.00	−0.50 (−0.89 to −0.15)^*^
Onset	256.9±63.6	245.7±42.4	−0.19 (−0.68 to 0.27)	222.5±30.7	246.7±34.3	0.71 (0.03 to 1.43)	242.6±42.9	253.0±47.6	0.22 (−0.44 to 0.89)
**C3**									
Amplitude	10.27±4.48	14.04±5.56	0.71 (0.10 to 1.37)^*^	12.67±4.29	11.44±3.92	−0.29 (−0.72 to 0.13)	13.02±4.00	11.50±3.71	−0.37 (−0.78 to −0.01)
Onset	224.7±28.9	222.8±32.9	−0.06 (−0.76 to 0.64)	232.1±33.2	219.8±34.1	−0.35 (−0.71 to −0.01)	219.1±32.9	229.2±34.1	0.29 (−0.12 to 0.70)

*The gray columns show the effect size and 95% confidence interval for the change in each of these waveform components for each condition (modulation, rest and exercise). Unless specified, all data relate to responses to moderately painful stimulation*.

* p < 0.05;

† *p < 0.01*.

### Comparison of SEP and LEP amplitude changes

Comparing the evoked potentials in each experiment, N2P2 onset latency was significantly later for LEPs in *Experiment 2* than SEPs in *Experiment 1* (*Experiment 1*: 119 ± 12 ms (mean ± SD); *Experiment 2*: 229 ± 34 ms; [*d* = 4.13 (2.95 to 5.50), *p* < 0.001]. A contrast of both experiments and the rest and exercise conditions using repeated measures ANOVA revealed a significant experiment × condition × time effect on N2P2 amplitude (*p* = 0.047). Based on the mean change scores, the influence of rest on the amplitude of the evoked potentials was not statistically different between experiments [*Experiment 1*: −20.7 ± 20.6% (mean ± SD); *Experiment 2*: −7.3 ± 21.8%; *d* = 0.61 (−0.09 to 1.33), *p* = 0.08, Figures 4E,F]. In contrast, the effect of exercise on the amplitude of SEPs and LEPs differed significantly and with a moderate-large effect size [*Experiment 1*: 10.9 ± 44.6% increase (mean ± SD); *Experiment 2*: 14.6 ± 16.0% decrease; *d* = −0.74 (−1.48 to 0.04), *p* = 0.04; Figures 4C,D].

### Pain and anxiety ratings

**Figure 5:**
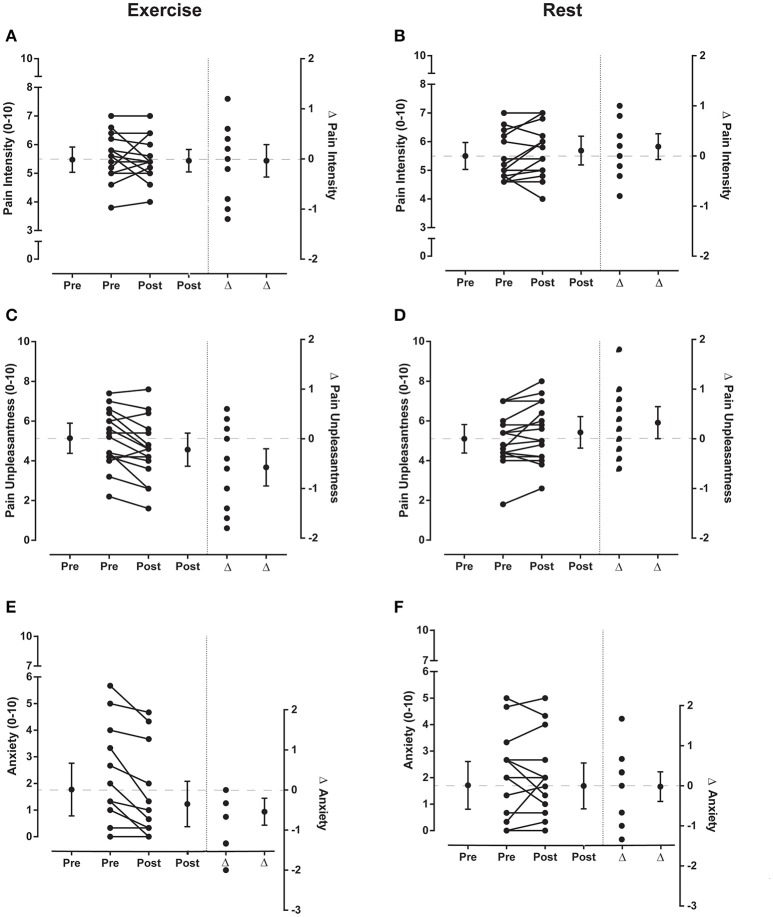
**Pain ratings for electrical stimuli ***Experiment 1*****. Individual and group data (mean and 95% confidence interval) for ratings of pain intensity, pain unpleasantness and anxiety (left side of vertical dotted lines in each graph) before (pre) and after (post) exercise (left panels) or rest (right panels) during *Experiment 1*. Five ratings were averaged to give a single value for ratings of pain intensity and pain unpleasantness for the sets of electrical stimuli and 3 ratings were averaged to give a single value for anxiety. Individual and group differences (Δ; mean and 95% confidence interval) in ratings from pre to post exercise or rest are shown to the right side of the vertical dotted line in each graph. In each of these plots the zero-difference level on the right-hand y-axis is aligned to the group mean for the pre-exercise reference condition. Data to the left of the vertical dotted line are plotted against the left-hand y-axis and data to the right of the vertical dotted line are plotted against the right-hand y-axis.

For electrical stimulation, there was no significant effect of time, condition, nor a time × condition interaction for ratings of pain intensity (all *p* > 0.12). There was no significant effect of time (*p* = 0.23) or condition (*p* = 0.07) for ratings of pain unpleasantness, but a significant time × condition interaction was observed [*F*_(1, 15)_ = 11.92, *p* = 0.004]. Ratings of pain unpleasantness were significantly lower after exercise [*d* = −0.37 (−0.65 to −0.11), *p* = 0.01] but not quiet rest [*d* = 0.22 (0.001 to 0.45), *p* = 0.10; Figure 5]. For ratings of anxiety, there was a significant effect of time [*F*_(1, 15)_ = 6.73, *p* = 0.02] but not condition (*p* = 0.05) nor a time × condition interaction (p = 0.15). Ratings of anxiety were significantly lower after exercise (*d* = −0.30, −0.52 to −0.10, *p* = 0.004) but not after quiet rest [*d* = −0.01 (−0.22 to 0.19), *p* = 0.90; Figure 5].

For laser stimulation, there was no significant effect of time, condition, or a time × condition interaction for ratings of pain intensity, pain unpleasantness or anxiety (all *p* > 0.19). Hence, quiet rest had no effect on ratings (mean ± SD) of pain intensity [pre: 5.1 ± 1.6; post: 5.2 ± 1.4, *d* = 0.04 (−0.09 to 0.20)], pain unpleasantness (pre: 4.7 ± 2.1; post: 4.8 ± 2.0, *d* = 0.05 (−0.06 to 0.2)] or anxiety [pre: 2.1 ± 2.0; post: 1.9 ± 1.8, *d* = −0.06 (−0.18 to 0.05]. Similarly, exercise had no effect on ratings (mean ± SD) of pain intensity [pre: 4.9 ± 1.3; post: 5.0 ± 1.7, *d* = 0.07 (−0.25 to 0.41)], pain unpleasantness [pre: 4.8 ± 2.0; post: 4.8 ± 2.2, *d* = 0.04 (−0.15 to 0.23)] or anxiety [pre: 2.2 ± 2.1; post: 2.1 ± 2.2, *d* = −0.07 (−0.22 to 0.08)].

There were no significant differences between the experiments in the average ratings (mean ± SD) of pain intensity [*Experiment 1*: 5.5 ± 0.7; *Experiment* 2: 4.8 ± 1.3; *d* = −0.65 (−1.37 to 0.05), *p* = 0.07], pain unpleasantness [*Experiment 1*: 5.1 ± 1.3; *Experiment* 2: 4.5 ± 1.8; *d* = −0.34 (−1.04 to 0.35), *p* = 0.33] or anxiety [*Experiment 1*: 1.7 ± 1.6; *Experiment 2*: 2.0 ± 1.8; *d* = 0.15 (−0.54 to 0.85, *p* = 0.66)].

## Discussion

This novel investigation of the mechanisms by which exercise acutely relieves pain in healthy adults did not produce a straightforward result. The hypoalgesic effect of isometric exercise was clear in pressure pain thresholds but absent for heat pain thresholds. LEPs, the responses to painful heat stimuli, were significantly reduced after exercise and non-significantly reduced after a similar period of rest. SEPs in response to painful electrical stimuli were unchanged after exercise but significantly reduced after a similar period of rest. Finally, neither exercise nor rest changed ratings of pain intensity for either the electrical or laser stimuli, but exercise did reduce ratings of pain unpleasantness and anxiety for the electrical stimuli only. Thus, we found apparent inconsistencies in the effect of exercise on EEG and perceptual responses, as well as on different modalities of pain.

### Verifying the acute hypoalgesic effect of exercise

A similar and substantial hypoalgesic effect of exercise in both experiments was verified by the elevation of pressure pain thresholds (PPTs) over the exercised muscle (biceps brachii) and elsewhere (first dorsal interosseous (FDI)). The larger increases in PPTs at biceps brachii than FDI were consistent with previous reports of greater increases in PPT for exercised compared to non-exercised limbs or muscles (Koltyn and Umeda, 2007; Naugle et al., 2014b; Vaegter et al., 2014). Our measurement of PPT directly over the muscle, including the FDI, contrasted with the application of electrical stimuli to the index finger (*Experiment 1*) or laser stimuli to the dorsum of the hand (*Experiment 2*) for the evoked potentials. However, other investigators have reported similar large increases in pain threshold to mechanical pressure applied over bones of the finger, confirming that the hypoalgesic effect of exercise is not confined to nociceptive input from muscles (Koltyn and Umeda, 2007; Hoeger Bement et al., 2008).

Although pain thresholds were not re-evaluated during or immediately following the measurement of evoked potentials, it is well-established that EIH endures for at least 10 min after exercise (Persson et al., 2000; Naugle et al., 2012). This duration of EIH would have spanned the approximately 7-min for the evoked potential recordings, which commenced within 1–2 min of the exercise and immediately following the pain threshold measurements. Pain thresholds were measured again 30 min after the exercise to verify that by that time the EIH had dissipated. Measurement of MVCs at the beginning of the experiment also preceded the initial measurement of evoked potentials and pain thresholds by at least 30 min to ensure that there was no effect of EIH from this initial muscle activity.

The slight difference in RPE between experiment 1 and 2 most likely arose from the greater number of female participants in experiment 1. Females are typically more resistant to muscle fatigue during sustained isometric contractions performed at fixed proportions of maximal strength (Hunter, 2014). The small difference in average RPE did not appear to impact the extent of EIH between the experiments.

### Habituation of the evoked potentials

Habituation of evoked potentials, thought to involve both peripheral and central components (Hüllemann et al., 2013, 2015), has previously been reported (Greffrath et al., 2007; Smith et al., 2008; Hüllemann et al., 2013) and was not an unexpected finding in either of our experiments. It is, however, an obvious limitation in the utility of evoked potentials to explore the mechanisms of EIH and needs to be addressed in future studies that use these techniques. The inclusion of a rest condition was an important component of our experiment design. It identified how much change in evoked potential amplitude arose from habituation alone (i.e., approximately 21% for SEPs and approximately 7% for LEPs) as a basis to carefully interpret any observed changes with exercise.

### Modulation of the evoked potentials

The measured N2P2 components of the evoked potentials were larger when stimulus intensity was increased and the stimuli were rated as more painful. This modulation condition of the experiments verified that the evoked potentials scaled with stimulus intensity and were sensitive to change. Evoked potential amplitude is affected by stimulus intensity, analgesics, mood and attention (Vossen et al., 2006; Wang et al., 2010; Hoeben et al., 2012; Hu et al., 2014; Castro et al., 2015), suggesting that both the nociceptive/affective and evaluative/functional aspects of pain are represented. We ensured that all key influences—such as anxiety, attention to the noxious stimulus and electrode impedance—were held constant throughout the experiment. Hence, these factors are unlikely to have influenced the results.

### Minimal reduction of the laser evoked potentials

Consistent with our hypothesis, LEPs were reduced after exercise. This finding should be interpreted with caution because the effects of rest and exercise on LEP amplitude were not significantly different. The minor influence of exercise on the LEPs may arise from the limited influence of exercise on heat pain, in addition to these measures being distant from the exercised muscle. EIH is known to be larger and more consistent when mechanical rather than thermal stimuli are used to evoke pain (Koltyn, 2000; Naugle et al., 2012; Vaegter et al., 2016). This is supported by the current study as the effect of exercise on heat pain thresholds (HPTs) was absent compared to that on PPTs. There are several possible explanations for this. First, HPT was not assessed at the primary exercised muscle where EIH would have been greatest. Second, in order to equate the method of measuring heat pain with that of pressure pain, we stimulated a much smaller surface area and more rapidly increased heat than previous studies (Kodesh and Weissman-Fogel, 2014; Coronado et al., 2015). Third, EIH is larger and more consistent when mechanical rather than thermal stimuli are used to evoke pain (Koltyn, 2000; Hoffman et al., 2004; Ruble et al., 2005; Naugle et al., 2012; Vaegter et al., 2016), but exactly why this occurs is not known. It is possible that evoking EEG potentials using a painful mechanical stimulus (Iannetti et al., 2013; Van den Broeke et al., 2015) may detect greater change with exercise than was detected using LEPs. However, mechanically evoked potentials remain poorly understood with regard to the component(s) of these potentials that are most associated with the activity of nociceptive pathways. It is also possible that a greater dose of exercise may have elicited more EIH and had a bigger influence on HPT and LEP amplitude.

A further consideration for the comparative effects of exercise on LEPs and pain threshold measures is that these responses may be subserved by different classes of nociceptive afferents which exercise may affect differentially. The latency of the LEP N2P2 component was consistent with activity of A-delta fibers. The activation of nociceptors by heat from a laser requires energy transmission to the receptor followed by transduction of this energy and action potential generation (Treede et al., 1995). A-delta fibers respond within approximately 40–100 ms of the rapid application of heat energy, depending on the stimulus intensity and the proximity of the laser beam to the receptive field of the nociceptor (Xu et al., 2008; Zhu and Lu, 2010). Thus, the approximately 230-ms onset latency of the N2P2 component of the LEP was consistent with the combination of the stimulus transduction time and the conduction velocity of A-delta fibers. Responses to brief punctate noxious stimuli (i.e., pinpricks) are conveyed by A-delta fibers (Dubin and Patapoutian, 2010), however our assessment of PPTs occurred over a period of seconds and elicited a sensation of gradually increasing blunt pressure and then pain. This is obviously different to a pinprick sensation and it is possible that pain thresholds to this slower application of pressure are associated more with activity of C-fibers. Hence, if exercise has greater effects on C- compared to A-delta fibers, this might explain why PPTs changed after exercise much more so than LEPs, but this is speculative.

### Negligible change of the somatosensory evoked potentials

Habituation makes it difficult to conclude whether exercise genuinely had no effect on SEP amplitude or simply alleviated the effect of quiet rest. It is even possible that exercise led to increased signal from some generators of the SEP and that this was superimposed on habituation, resulting in no net effect. Despite a similar degree of habituation following rest, the SEP and LEP responses to exercise clearly differed. Several factors could explain the different effect of exercise on the SEP and the LEP.

An inherent limitation of comparing SEPs and LEPs is the different neural pathways engaged by each type of stimulus and the extent to which these reveal nociceptive activity. LEPs and SEPs are often compared to distinguish changes in nociceptive pathways, such as assessing the spinothalamic tract (LEPs) vs. non-nociceptive sensory pathways like the dorsal column-lemniscal system (SEPs) (de Tommaso et al., 2011; Perchet et al., 2012). However, such comparisons typically involve the early components of the SEP whereas in the current study, the late N2P2 response to painful electrical stimulation was measured. The so-labeled ‘noxious component’ of the SEP involves the activation of A-delta fibers (Dowman and Bridgman, 1995), shares common cortical and subcortical generators with the equivalent component of the LEP (Cruccu et al., 2008), and has been shown in several investigations to be reduced by analgesic medication (Bromm et al., 1986; Kochs et al., 1996).

In the current study, the SEP scaled with the intensity and rating of noxious electrical stimulation. Notably, the stimulus intensity to elicit mild pain was approximately 10x perceptual threshold and that to elicit moderate pain was approximately 14x perceptual threshold. Since both these intensities exceed the typical recruitment thresholds for large-fiber sensory cutaneous afferents, yet the peak-to-peak amplitude of the N2P2 component of the SEP increased markedly (Figure 4A), a contribution from the higher threshold, thinly myelinated A-delta fibers seems likely. Indicative of saturation in the large diameter afferent contribution to the EEG, the N1 potential amplitude did not increase from the mild-pain to moderate-pain stimulation intensities, which provides further support for the A-delta contribution to the N2P2 measure. Nonetheless, the possibility cannot be excluded that the electrically-evoked N2P2 was insensitive to the analgesic effects of exercise because of the contribution of non-nociceptive pathways to the SEP. Notably, the influence of exercise and rest on N1 potential amplitude was similar to that for the N2P2 component of the SEP.

Another possible reason for the absence of a reduction in the SEP amplitude or the rating of pain intensity with exercise is that the electrical activation of nerve axons in the index finger may have been inherently less sensitive to EIH by not involving the peripheral nociceptors. The approximately 120 ms onset latency of the N2P2 component of the SEP was consistent with the direct activation of the A-delta afferent axons for the electrically-evoked response (i.e., approximately 8 ms^−1^ for the fastest of the afferents contributing to the N2P2). Though we did not measure nociceptor activity directly, a change in excitability of the peripheral nociceptors in response to exercise is physiologically plausible; nociceptive primary afferents can be modulated via receptors at the periphery (Carlton, 2014), and many of the substances that influence nociceptor sensitivity are increased in the blood during exercise (e.g., opioids and catecholamines; Galbo et al., 1975; Thorén et al., 1990; de Souza et al., 2013).

There were also differences in the stimuli for SEPs and LEPs other than the involvement or not of peripheral nociceptors. For example, the electrical stimuli were more frequent, far briefer, and would have evoked less temporally dispersed volleys than the heat stimuli. Thus, some possible explanations for the different behaviors of the SEP and LEP after exercise are that exercise directly affected the nociceptors, or that the different stimulus profiles may have been differently sensitive to spinally-mediated inhibitory control mechanisms.

### Ratings of pain intensity, unpleasantness and anxiety

Despite the reduction in LEP amplitude, there was no effect of exercise (or rest) on the rating of pain intensity, pain unpleasantness or anxiety with the brief infrequent laser heat stimuli. Previous studies showing an effect of exercise on ratings of pain intensity have used a contact thermode to deliver a continuous (30-120 s) heat stimulus to participants who rated their pain every 5–10 s (Kodesh and Weissman-Fogel, 2014; Naugle et al., 2014b). This continuous method may provide participants with a greater ability to discern between different intensities of heat. For the electrical stimulus, there was no effect of exercise on ratings of pain intensity. This was consistent with no reduction in SEP amplitude, but is in contrast to previous reports of reduced ratings of pain intensity (Naugle et al., 2012; Micalos et al., 2015; Umeda et al., 2016b). Again, this might be due to methodological differences between our study and past investigations.

While ratings of pain intensity were unaffected, ratings of anxiety and pain unpleasantness of the electrical stimuli were both reduced after exercise. These findings suggest that EIH may involve a central effect on higher order psychological processes. A previous investigation from our laboratory demonstrated that aerobic training increased tolerance of a noxious ischemic stimulus independent of a change in its perceived intensity (Jones et al., 2014) and cross-sectional studies have also found that athletes are more tolerant of pain despite having similar pain thresholds to non-athletes (Tesarz et al., 2012). The results of *Experiment 1*, as well as those of past studies (Umeda et al., 2010; Ellingson et al., 2014; Vaegter et al., 2016), show that acute exercise can exert a similar effect and suggest that changes in pain appraisal contribute to EIH.

## Conclusion

The novel application of neurophysiological techniques has highlighted that changes in central and peripheral areas of the nervous system might underlie EIH in healthy adults. Changes in ratings of pain unpleasantness and anxiety during SEP recordings, without a change in pain intensity, support a centrally-mediated influence of exercise. The different behavior of the somatosensory evoked potentials to the laser evoked potentials following exercise could indicate that peripheral nociceptors contribute to exercise-induced hypoalgesia. However, the effect of exercise on the amplitude of SEPs and LEPs was negligible when compared to the change observed with quiet rest (i.e., habituation). Overall, the small or absent changes in the N2P2 component of the evoked potentials suggest a minor influence of exercise on A-delta pathways although there are substantial changes in pressure pain threshold, but not heat pain threshold.

Investigations using techniques that can directly isolate and examine these subsections of the nociceptive pathways are needed to determine exactly where the changes might be occurring. With regard to the utility of EEG evoked potentials for this purpose, this will be restricted by habituation of these potentials, it may require the measurement of potentials in response to painful mechanical stimuli, and could benefit from the application of protocols to record C-fiber responses in addition to A-delta responses.

## Ethics statement

All procedures were approved by the University of New South Wales Human Research Ethics Committee (HC 14065) and conformed to the requirements of the Declaration of Helsinki (2008). Written informed consent was obtained from each participant prior to testing.

## Author contributions

MJ, JT, JB, and BB conceived and designed the experiments; MJ, BB performed the experiments and analyzed the data; MJ, JT, and BB interpreted the results; MJ, BB drafted the manuscript; MJ, JT, JB, and BB revised the manuscript critically for important intellectual content. MJ, JT, JB, and BB approved the final version of the manuscript.

## Funding

Support for this work was provided by a Program Grant (1055084) from the National Health and Medical Research Council of Australia (NHMRC). MJ holds an Australian Postgraduate Award and JT holds a NHMRC Research Fellowship.

### Conflict of interest statement

The authors declare that the research was conducted in the absence of any commercial or financial relationships that could be construed as a potential conflict of interest.
